# Mapless mobile robot navigation at the edge using self-supervised cognitive map learners

**DOI:** 10.3389/frobt.2024.1372375

**Published:** 2024-05-22

**Authors:** Ioannis Polykretis, Andreea Danielescu

**Affiliations:** Accenture Labs, San Francisco, CA, United States

**Keywords:** navigation, planning, autonomous, robot, edge, self-supervised, local learning, neuromorphic

## Abstract

Navigation of mobile agents in unknown, unmapped environments is a critical task for achieving general autonomy. Recent advancements in combining Reinforcement Learning with Deep Neural Networks have shown promising results in addressing this challenge. However, the inherent complexity of these approaches, characterized by multi-layer networks and intricate reward objectives, limits their autonomy, increases memory footprint, and complicates adaptation to energy-efficient edge hardware. To overcome these challenges, we propose a brain-inspired method that employs a shallow architecture trained by a local learning rule for self-supervised navigation in uncharted environments. Our approach achieves performance comparable to a state-of-the-art Deep Q Network (DQN) method with respect to goal-reaching accuracy and path length, with a similar (slightly lower) number of parameters, operations, and training iterations. Notably, our self-supervised approach combines novelty-based and random walks to alleviate the need for objective reward definition and enhance agent autonomy. At the same time, the shallow architecture and local learning rule do not call for error backpropagation, decreasing the memory overhead and enabling implementation on edge neuromorphic processors. These results contribute to the potential of embodied neuromorphic agents utilizing minimal resources while effectively handling variability.

## 1 Introduction

The navigation of mobile agents in unfamiliar environments is a crucial first step for autonomously accomplishing progressively complicated tasks. Global knowledge of the environment, typically in the form of meticulously constructed maps, contains all the information required for effective navigation through efficient planning (Meyer and Filliat, 2003). Although such global knowledge dramatically simplifies the problem, generating and storing these maps has significant resource demands (Egenhofer, 1993). Long mapping sessions or their supervised formation limit the applicability of such methods for consumer-oriented agents, whose ease of deployment and use is of central importance. In large-scale environments such as those encountered during rescue missions (Niroui et al., 2019), and planetary (Schuster et al., 2019) or underwater explorations (Rosenblatt et al., 2002), creating such maps is practically infeasible and actual autonomy is crucial. Lastly, dynamic environments limit the usefulness of a static map, while its real-time update introduces additional computational complexity.

To bypass the acquisition and storage of global knowledge of the environment, current planning methods aim to utilize limited global cues and combine them with local sensory information about the agent and its immediate surroundings (Tai et al., 2017; Zhu et al., 2017; Tang et al., 2020; Ding et al., 2022). Integrating the core principles of such methods with the learning capabilities inherent in modern Deep Neural Networks (DNNs) and the recent advancements in Reinforcement Learning (RL) has paved the way for achieving optimal solutions (de Jesús Plasencia-Salgueiro, 2023). However, achieving optimality with Deep Reinforcement Learning (DRL) solutions requires time, computing resources and power, which are not readily available in edge solutions.

The first source of resource requirements in DRL is the utilization of multi-layer networks to exploit their escalating computational capacity when handling high-dimensional problems. However, such architectures require training with error backpropagation, the backbone of DL (Rumelhart et al., 1986). While adaptable to virtually any task with remarkable effectiveness, backpropagation is not yet universally applicable to edge hardware. Deep networks (Chowdhery et al., 2019) and ensemble models (Vergara et al., 2012) based on backpropagation have been successfully deployed in microprocessors, but their implementation on neuromorphic processors (Furber et al., 2014; Merolla et al., 2014; Moradi et al., 2017; Davies et al., 2018; Pehle et al., 2022) that promise even lower power consumption is challenging. Even when successfully adapted to neuromorphic hardware (Neftci et al., 2017; Renner et al., 2021), backpropagation introduces additional space complexity during both the training and inference phases, posing memory footprint challenges (Chishti and Akin, 2019). In contrast, neuromorphic processors draw their efficiency from utilizing local learning rules that update a few parameters without necessitating data transfer (Burr et al., 2017; Zenke and Neftci, 2021), highlighting the need for algorithms leveraging local learning.

The second origin of resource requirements is the meticulously tailored reward objectives required for DRL, which make extensive training sessions and careful tuning imperative. Influential methods from DRL for policy learning (Schulman et al., 2015; Schulman et al., 2017), Q learning (Mnih et al., 2013), or their combination (Mnih et al., 2016; Haarnoja et al., 2018) have demonstrated remarkable results in navigation tasks (de Jesús Plasencia-Salgueiro, 2023). However, such methods require the precise definition of reward objectives adapted to the given task, and result in the need for extensive training sessions and significant tuning. In contrast, autonomous agents at the edge can benefit significantly from self-supervision strategies (Kahn et al., 2018; Kahn et al., 2021) that would allow on-chip training to utilize minimal resources.

This work leverages the computational advantages of combining a self-supervised approach with local learning rules on edge hardware. We adapted Cognitive Map Learners (CML) (Stöckl et al., 2022), a brain-inspired planning method that has been applied to hierarchical action selection (McDonald, 2023), to a continuous 2D navigation task. We did so by minimizing the model’s embedding space to only encode the agent’s position and its action space to a few discrete actions to support navigation. We also used more elegant exploration strategies to push the navigation performance closer to the optimal standards. Our approach performs comparably to the RL baseline (DQN) (Mnih et al., 2013) on goal-reaching accuracy and path optimality while necessitating a similar number of parameters, operations, and training iterations. Notably, our self-supervised method not only enhances agent autonomy but also benefits from a shallow architecture and a local learning rule, mitigating the necessity for backpropagation.

In summary, our main contributions are as follows.

• The adaptation of CML to a continuous 2D navigation task while minimizing the model’s dimensions and, consequently, its resource requirements,
• The enrichment of the model’s training with elegant exploration strategies that brought its performance closer to optimality,
• The extension of the method and its evaluation in dynamic environments with unmapped obstacles.

Our results highlight the fitness of our approach for learning directly on neuromorphic processors, aligning with the overarching goal of embodied neuromorphic agents exhibiting robust performance with minimal resource utilization at the edge.

## 2 Methods

### 2.1 CML network architecture

**FIGURE 1:**
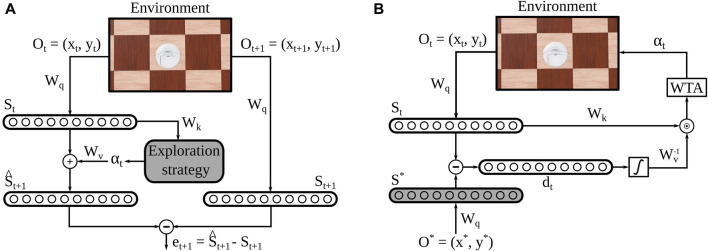
Cognitive Map Learner Network Architecture. **(A)** During each training step, the state of the agent *o*_*t*_ is embedded into a high-dimensional vector *s*_*t*_. Then, an action is taken based on some exploration strategy, and its effect on the embedded state is estimated $\left({\hat{s}}_{t+1}\right)$ . Based on the difference *d*_*t*_ between this estimate and the actual next embedded state *s*_*t*_, the network parameters *W*_*q*_, *W*_*k*_ and *W*_*v*_ are optimized. **(B)** During inference, the embedded agent’s state *s*_*t*_ is compared with a desired embedded state s*. Based on the difference *d*_*t*_ between these and the allowed actions at the current state given by *W*_*k*_, a Winner-Take-All (WTA) unit selects the most beneficial allowed action *α*_*t*_ for the agent to perform.

Cognitive Map Learners (CML) are a recently proposed architecture comprising of three distinct yet collaboratively trained, single-layer, artificial neural networks. The three networks can be trained to learn and utilize high-dimensional representations of the nodes and edges in arbitrary bidirectional graphs. With the graph nodes encoding the states of a system and the edges encoding actions that lead to the transition between states, CML can learn the system’s internal dynamics. The CML utilized in this paper adhere to the design principles outlined by Stöckl et al. (2022) in the original paper introducing this network architecture (Figure 1). The CML operates in two distinct modes: training and planning. In the training mode (Figure 1A), the CML explores the environment using a predefined strategy. In the planning mode (Figure 1B), the network uses its learned architecture to plan a sequence of actions to get from an initial state to a desired goal state. In this work, we adapted the method proposed in the original paper to a 2D navigation task without changing its core features. We also used the proposed random exploration strategy during training, but experimented with a more elegant strategy to examine whether it would improve the method’s performance in our navigation task. Our strategy included a novelty-based exploration factor (see Section 2.2) to promote the faster visitation of unseen locations in larger environments.

At each exploration step, the agent collects an observation of its environment $o_{t}=\left(x_{t},y_{t}\right)\in R^{N_{i}}$ , where *x*_*t*_, *y*_*t*_ are the coordinates of the agent on a 2D plane and, therefore, the dimension of the observation space is *N*_*i*_ = 2. Then, it creates a high-dimensional state space embedding $s_{t}\in R^{N_{s}}$ , where *N*_*s*_ is the dimension of the embedding space. Notably, we do not digitize the locations into a 2D discrete grid, but we directly feed the continuous values of the spatial coordinates into the embedding of the network. In that way, the continuous state space (spatial coordinates and their embedding) resulted in a method complexity that does not scale proportionally to the size of the environment (number of required grid nodes).

After that, the agent takes an action *α*_*t*_ ∈ *N*_*α*_, where *N*_*α*_ is the dimension of the action space. The structure of the original CML model requires a discrete action space because the action selection is realized through a discrete Winner-Take-All mechanism. With our choice of 8 possible actions, we provided the method with a base of actions that were able to effectively drive the agent to the required environment locations in the 2D plane without complicating the model architecture.

Then, the agent produces an estimate of its next state ${\hat{s}}_{t+1}\in R^{N_{s}}$ based on the action taken and the current state. The agent then supervises its own performance by calculating a training error $\left|s_{t+1}-{\hat{s}}_{t+1}\right|$ , defined as the distance between its estimated next state and the actual observed state. Using a local, self-supervised learning rule, the agent computes an update of its CML architecture, which comprises three matrices: *W*_*q*_, *W*_*k*_, and *W*_*v*_. The matrix $W_{q}\in R^{N_{s}\times N_{i}}$ embeds state observations into a high-dimensional space; $W_{k}\in R^{N_{\alpha }\times N_{s}}$ maps state embeddings *s*_*t*_ to affordance values *g*_*t*_ = *Sigmoid* (*W*_*k*_ *s*_*t*_), which estimate whether an action is available at the current state. Lastly, $W_{v}\in R^{N_{s}\times N_{\alpha }}$ maps actions to estimates of their potential impact on the agent’s state. After each update calculation, this process is repeated for a defined number of steps, constituting a training episode, during which the matrix updates accumulate. At the end of each episode, the matrix updates are applied to optimize the CML architecture, as shown in the Eqs 1–3 below:

$$W_{k}=W_{k}+\overset{T}{\underset{t=1}{\sum }}\Delta W_{k}^{t},\;\;\Delta W_{k}^{t}=\ell_{k}\left(\alpha_{t}-g_{t}\right)s_{t}^{T},$$ (1)

$$W_{v}=W_{v}+\overset{T}{\underset{t=1}{\sum }}\Delta W_{v}^{t},\;\;\Delta W_{v}^{t}=\ell_{v}\left(s_{t+1}-{\hat{s}}_{t+1}\right)a_{t}^{T},$$ (2)

$$W_{q}=W_{q}+\overset{T}{\underset{t=1}{\sum }}\Delta W_{q}^{t},\;\;\Delta W_{q}^{t}=\ell_{q}\left({\hat{s}}_{t+1}-s_{t+1}\right)o_{t+1}^{T},$$ (3)

where $\Delta W_{i}^{t},i\in \left\{k,v,q\right\}$ are the matrix updates computed after each episode step, and *ℓ*_*i*_, *i* ∈ {*k*, *v*, *q*} are the learning rates for each matrix. Throughout this work, we set all three learning rates to 0.001, following the values used in (Stöckl et al., 2022).

After an arbitrarily chosen number of 10 episodes constituting en epoch, the trained model is evaluated by solving a planning task. The planning error *e*_*pos*_ = |*p*_*goal*_ − *p*_*final*_|, defined as the distance between the goal and final positions of the agent, serves as a validation metric.

To solve the planning task, the agent externally receives the goal location *p*_*goal*_ and embeds it into the state space using *W*_*q*_. The CML then utilizes the inverse of *W*_*v*_ to compute a utility score for each action, indicating their usefulness for reaching the goal state. In fact, in this work we exploited the orthogonal property of *W*_*v*_ the fact that *W*_*v*_ approximately behaves as an orthonormal matrix to avoid the matrix inversion and substitute it with a simple transpose operation (see Appendix in (Stöckl et al., 2022) for a detailed explanation.) Intuitively, the inverse of *W*_*v*_ can be sufficiently approximated by its transpose because the equation $u_{t}=W_{v}^{-1}d_{t}$ that computes the utility of each action can be well approximated by the inner products between the target vector *d*_*t*_ and the vectors encoding the impact of an action on the state space, which are the columns of *W*_*v*_. With the inner product being a similarity metric, this would result in higher utility scores for increasingly similar actions, which is the desired outcome. Concurrently, the CML uses *W*_*k*_ to estimate an affordance score for each action in the current state. These scores are combined through element-wise multiplication, and the most useful action among the affordable ones is selected in a Winner-Take-All (WTA) fashion. This process continues until either the agent reaches the goal location or a predefined maximum number of actions has been taken.

### 2.2 Novelty-based exploration policy

**FIGURE 2:**
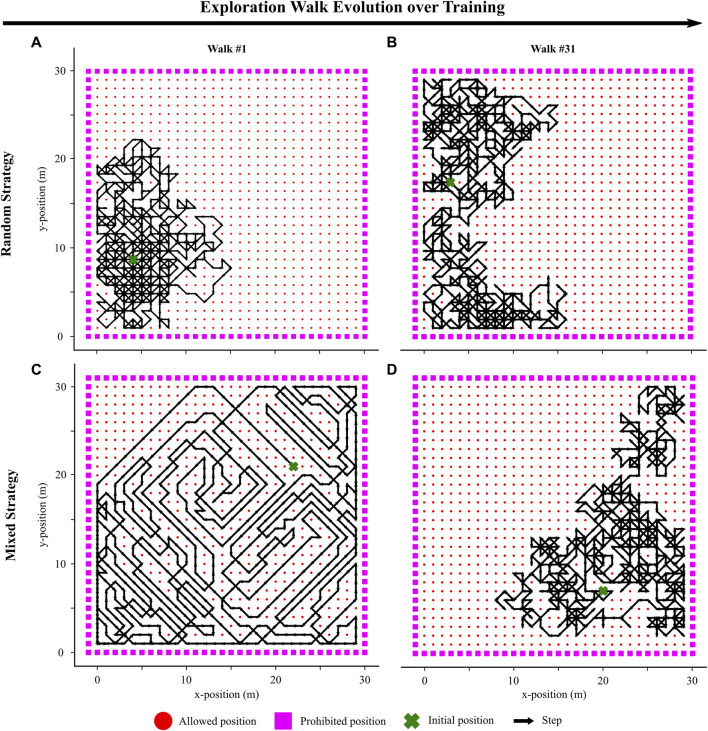
Visualization of two exploration walks in the environment with two different strategies. **(A)** Visualization of the 1st walk with a purely random exploration strategy. **(B)** Visualization of the 31st walk with the strategy being consistently purely random. **(C)** Visualization of the 1st walk with a mixed exploration strategy. The walk is purely novelty-driven. **(D)** Visualization of the 31st walk with a mixed exploration strategy. The walk is now purely random as the novelty-based effect has completely faded (Eq. 5).

To allow for our agent to explore and learn its environment, we started by implementing the random exploration strategy outlined in (Stöckl et al., 2022). At each timestep during exploration, the agent randomly selected to move in one of the eight possible directions in the 2D grid (Figures 2A,B). This strategy led to significant repetition of actions in the same locations and resulted in effective learning of local navigation policies. However, the randomness inherent in this strategy constrained the exploration of previously unseen parts of the environment. To investigate whether a more sophisticated approach could enhance navigation performance, we introduced a mixed exploration strategy by incorporating a novelty-based factor into the random steps.

In this mixed strategy, we defined a commonly used novelty metric (Tao et al., 2020), as follows:

$$\hat{\rho }\left(x\right)=\overset{k}{\underset{i=1}{\sum }}d_{Eu}\left(x,n_{i}\right).$$ (4)

The metric of Eq. 4 quantified the Euclidean distance *d*_*Eu*_ between a location *x* and its k nearest neighbors *n*_*i*_ in the set of previously visited locations. In our experiments, we set *k* to 5. Instead of selecting actions entirely at random, the exploration strategy prioritized actions that moved the agent to locations that maximized this novelty metric, aiming to emphasize visits to new, unseen parts of the environment.

However, the balance between the novelty-driven visitation of unseen locations and the effective learning of local navigation dynamics from the random exploration strategy is crucial. To preserve this balance, we gradually diminished the impact of the novelty-based factor in the first *N*_*expl*_ exploratory walks, transitioning to a purely random strategy in the latter walks. To balance the two strategies in the first *N*_*e*_ walks, we defined an exploration threshold *t*_*expl*_ as follows:

$$t_{\mathrm{expl}}=\left\{\begin{array}{cc} 1-\left(1/N_{\mathrm{expl}}\right)\;*\;i\; & i\leq N_{\mathrm{expl}}\\ 0\; & i>N_{\mathrm{expl}} \end{array}\right.$$ (5)

and chose the agent’s action at each timestep by comparing it with *s* ∼ *U* (0, 1). If the sampled value *s* fell below the threshold *t*_*expl*_, the following action was chosen based on the novelty metric; otherwise, the agent selected an action randomly. As the exploration threshold decreased to zero after the initial *N*_*expl*_ walks, a purely random strategy was employed during the remaining walks. In our experiments, we set *N*_*expl*_ to 0.3 of the total number of 100 exploratory walks.

We designed this mixed exploration strategy to guide the agent through a progression—from a purely novelty-based first walk (Figure 2C), gradually incorporating random actions during the first *N*_*expl*_ trials, to concluding with a purely random strategy for the remaining walks (Figure 2D). This approach aimed to initially encourage exploring novel, remote locations before focusing on learning efficient local navigation by repeating actions at previously visited locations.

### 2.3 Reinforcement Learning baseline

In order to compare our approach against the state-of-the-art, we implemented a RL baseline to tackle the same navigation task. We evaluated their respective training requirements, parameter count, and navigation performance.

Given the discrete nature of our action space, we opted for a DQN architecture (Mnih et al., 2013) to learn the policy required for the navigation task. Our selection comprised the smallest effective fully connected architecture featuring two hidden layers with *N*_*h*1_ = *N*_*h*2_ = 64 neurons each. Two neurons in the input layer received the agent’s coordinates *o*_*t*_ as input to encode its position in the 2D grid, while the output layer comprised eight neurons representing all the possible actions *α*_*t*_.

To define the optimization objective for the RL method, we initially set the agent’s cumulative reward during each training episode as follows:

$$R_{\mathrm{simp}}=\overset{T_{\mathrm{epis}}}{\underset{t=1}{\sum }}-|p_{\mathrm{goal}}-p\left(t\right)|,$$ (6)

where *p*_*goal*_ is the goal location, *p*(*t*) is the location of the agent at timestep *t*, and *T*_*epis*_ is the number of timesteps per episode. Throughout our experiments we clipped *T*_*epis*_ to 100.

The DQN, driven by this reward, aimed to bring the agent closer to the goal location. However, this simplified reward structure exhibited two drawbacks. Firstly, it approached its maximum value even when the agent was close but not precisely at the goal, compromising precision in reaching exact goal locations. Secondly, it assigned the same reward for reaching the same final location via two paths of different lengths, disregarding the number of actions taken and thereby promoting sub-optimal solutions. To mitigate these limitations, we modified the reward as follows:

$$\begin{array}{ll} R_{\mathrm{tail}} & =\overset{T_{\mathrm{epis}}}{\underset{t=1}{\sum }}\left\{-|p_{\mathrm{goal}}-p\left(t\right)|\right\}+R_{\mathrm{goal}}-R_{\mathrm{path}},\\ R_{\mathrm{path}} & =max\left\{t-d_{Ch}\left(p_{\mathrm{init}},p_{\mathrm{goal}}\right),0\right\}, \end{array}$$ (7)

where *R*_*goal*_ is an additional factor that increased the reward when the agent reached the actual goal location (we arbitrarily set it to 100), and *R*_*path*_ is a penalty factor that reduced the reward when the agent took more steps than the minimum required to reach the goal location. The factor *R*_*path*_ was set to zero when the number of actions taken was less than or equal to the minimum required (*t* < *d*_*Ch*_(*p*_*init*_, *p*_*goal*_)), while it became positive when more actions were taken (*t* ≥ *d*_*Ch*_(*p*_*init*_, *p*_*goal*_)). The amount *d*_*Ch*_(*p*_*init*_, *p*_*goal*_) denotes the Chebyshev distance between the initial location *p*_*init*_ and the goal location *p*_*goal*_ and quantified the minimum number of required actions to take the agent from the starting location to the goal location.

This refined reward structure addressed precision issues near the goal and incentivized the RL model to discover more efficient paths, improving the overall navigation performance. However, this required additional training. While we trained the DQN with the simplified reward for 100,000 timesteps to allow for convergence, the tailored reward required 200,000 total timesteps (Figure 4A). For all other parameters, we followed the already tuned implementation from (Hill et al., 2018).

### 2.4 Experiments and data analysis

#### 2.4.1 Performance evaluation metrics

To compare the performance of the CML method against the DQN baseline, we employed two metrics. Firstly, we evaluated the final position error *e*_*pos*_ of each method during planning, represented by the Euclidean distance between the agent’s final position *p*_*final*_ when driven by each planning method and the designated goal position *p*_*goal*_:

$$e_{\mathrm{pos}}=\|p_{\mathrm{goal}}-p_{\mathrm{final}}{\|}_{2}$$ (8)

The metric of Eq. 8 provided insights into the accuracy and precision of the planned trajectories in reaching the specified goal locations.

Secondly, we quantified the path overhead ratio *λ* for each planning method, as follows:

$$\lambda =\frac{S^{i}\left(p_{\mathrm{init}},p_{\mathrm{goal}}\right)}{d_{Ch}\left(p_{\mathrm{init}},p_{\mathrm{goal}}\right)},\;\;i\in \left\{CML,DQN\right\},$$ (9)

where *S* ^*i*^(*p*_*init*_, *p*_*goal*_) denotes the number of steps taken by method *i* when planning the navigation from *p*_*init*_ to *p*_*goal*_, and *d*_*Ch*_(*p*_*init*_, *p*_*goal*_) denotes the Chebyshev distance between the two locations on the 2D grid, which is also the minimum number of steps required to go from *p*_*init*_ to *p*_*goal*_. This metric measured the additional steps each planning method took compared to the optimal path required to reach a goal location from an initial position. The path overhead ratio offered a measure of efficiency, indicating the extent to which each planning method deviated from the most direct and optimal route.

#### 2.4.2 Hyper-parameter evaluation

To assess the impact of hyper-parameter choices on the navigation performance of the CML method, we conducted a series of experiments with varying values. Specifically, we manipulated the training parameters of the CML by altering the total number of walks and the number of steps per walk. In the first set of experiments, we fixed the number of walks at 100 and varied the number of steps per walk between 900, 600, 300, and 100. Subsequently, we set the number of steps per walk to 900 and adjusted the number of walks from 100 to 75, 50, and 25. The results of different hyper-parameter choices on the navigation performance of the CML are elaborated in Section 3.2.1.

Then, we varied the environment in which we tested the CML method. More specifically, we first examined the generalization ability of the CML when evaluated in larger environments than the ones in which it was trained. For this, we trained 10 CML models in 30 × 30 m arenas, then deployed them in 60 × 60 m and 120 × 120 m arenas. We evaluated their performance using the mean final position error and the mean path overhead ratio of the ten models (Section 3.3). Second, we examined the ability of the CML method to adapt to dynamic environments by training the agent in an initially empty 30 × 30 m arena and then adding an increasing number of obstacles during planning. For this, we trained ten CML models in the empty arena and gradually added 100, 200, 300, and 400 obstacles blocking locations the agent could initially reach. We evaluated the performance of the ten models by averaging their final position error (Section 3.4). In this case, we did not evaluate the path overhead ratio as the primary goal was to find any path to the goal, even if it was sub-optimal.

#### 2.4.3 Parameter and FLOPs quantification

To compare the number of parameters of our model against the baseline, we first counted the DQN parameters. Given the dimension of the observation space *N*_*i*_, which provided the input to the network, the dimension of the action space *N*_*α*_ that matched the network’s output, and our choice of *N*_*h*1_ and *N*_*h*2_ neurons in each of the 2 hidden layers, the 4-layer, fully-connected architecture resulted in a total parameter count of $\left(N_{i},\times ,N_{h1},\;,+,1,\;,\times ,N_{i}\right)$ + (*N*_*h*1_×*N*_*h*2_ + (*N*_*h*1_ + *N*_*h*2_)) + (*N*_*h*2_×*N*_*α*_ + 1×*N*_*α*_) $=\left(N_{i},\times ,N_{h1},+,N_{h1},\times ,N_{h2},+,N_{h2},\times ,N_{\alpha },N_{i},+,N_{h1},+,N_{h2},+,N_{\alpha }\right)$ weights and biases. Our CML model is fully described by the three matrices $W_{q}\in R^{N_{s}\times N_{i}}$ , $W_{k}\in R^{N_{\alpha }\times N_{s}}$ , and $W_{v}\in R^{N_{s}\times N_{\alpha }}$ , and, therefore, its total parameter count is (*N*_*i*_ + 2*N*_*α*_) × *N*_*s*_.

To compare our model against the baseline based on the number of floating point operations (FLOPs) for one inference, we first counted the DQN FLOPs. Given the 4-layer, fully connected DQN architecture described above and the requirement for 2 × *N*_*x*_ × *N*_*y*_ FLOPs for a fully-connected layer with *N*_*x*_ input and *N*_*y*_ output units, the total FLOPs of the DQN per inference were 2 × *N*_*i*_ × *N*_*h*1_ + 2 × *N*_*h*1_ × *N*_*h*2_ + 2 × *N*_*h*2_ × *N*_*α*_. To calculate the FLOPs during an inference step of our CML model, we note that the multiplication of an *n* × *p* and a *p* × *m* matrices requires *nm* (2*p* − 1) FLOPs. Therefore, the embedding of the observed (*o*_*t*_) and the desired $\left(o_{t}^{*}\right)$ positions into the vector *d*_*t*_ through *W*_*q*_ requires (2*N*_*i*_ + 1)*N*_*s*_ FLOPs. Additionally, calculating the affordance scores *g* for the current position using *W*_*k*_ requires (2*N*_*s*_ − 1)*N*_*α*_ FLOPs. Moreover, the calculation of the utility scores for the current affordable actions through *W*_*v*_ also requires (2*N*_*s*_ − 1)*N*_*α*_ FLOPs. Lastly, the calculation of the most useful affordable actions requires *N*_*α*_ FLOPs. Therefore, one inference step requires (2*N*_*i*_ + 4*N*_*α*_ + 1)*N*_*s*_ − *N*_*α*_ FLOPs in total.

### 2.5 Simulation environment

While our method demonstrated effectiveness in the discretized grid scenario, our objective was to assess its performance in a continuous task, evaluating its capacity to manage the variability associated with real-valued location encoding. To do so, we opted for the navigation task involving a wheeled robot agent navigating a planar arena environment.

**FIGURE 3:**
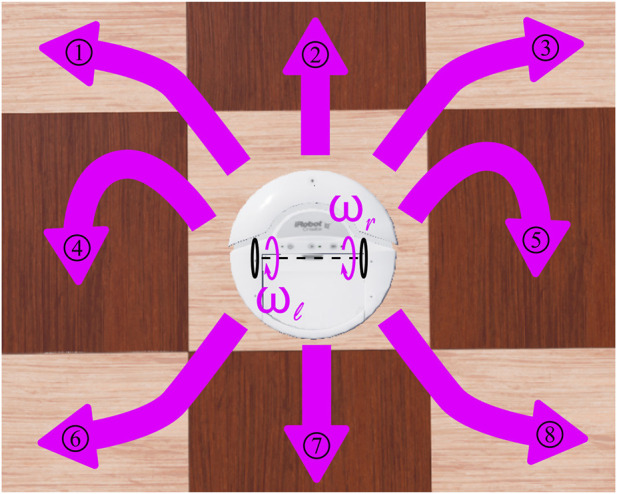
Possible directions of the robot motion constituting its discrete action space as a function of its wheels rotational velocities. The pairs of wheels rotational velocities that give rise to the respective numbered actions are given in Table 1.

To align with the discrete, 8-dimensional action space employed by the agent in the original 2D grid (Figure 3), we chose a set of eight pairs of wheel rotational velocities (Table 1) that drove the robot’s movement in the eight possible directions, similarly to the discrete case. After each action selection by the CML at the end of an episode step, the wheel velocity pair was applied to the robot for 64 simulation steps in *Webots* to move the robot.

**TABLE 1:** Wheel rotational velocities as a function of the maximum rotational velocity *ω*_*max*_ =5*π*.

Action #	*ω*_*ℓ*_/*ω*_*max*_	*ω*_*r*_/*ω*_*max*_
**1**	0.5	0.75
**2**	0.5	0.5
**3**	0.75	0.5
**4**	0.2	0.9
**5**	0.9	0.2
**6**	−0.75	−0.5
**7**	−0.5	−0.5
**8**	0.75	0.5

We used the Webots framework (Michel, 2004) for our simulations, an open-source mobile robot simulation software developed by *Cyberbotics Ltd*. We chose this platform because it has been successfully used by other research groups in prior work for simulating wheeled mobile robots (Tan et al., 2002; Almasri et al., 2015; Almasri et al., 2016). We chose *iRobot’s Create* robot prototype as our agent and a simple planar, 30 × 30 m arena as the environment, both included in this software package (Figure 9).

## 3 Results

### 3.1 Computational efficiency comparison against RL baseline

**FIGURE 4:**
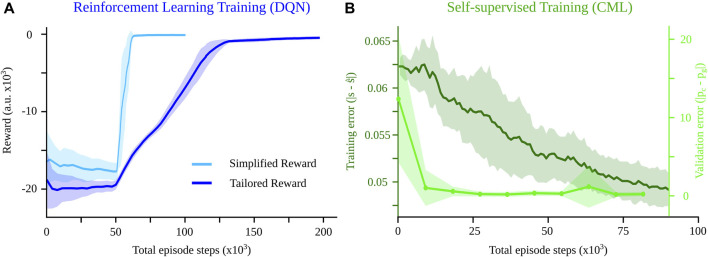
Comparison of the CML against the DQN baseline in terms of training speed. **(A)** While a simplified reward (equation 6) converges to a solution after about 60 K steps, a more sophisticated reward requires about 130 K. **(B)** The training error for the CML keeps decreasing after 90 K steps, but the validation error converges much faster (20 K steps), comparable to the RL baseline. Shaded regions show the variance of the training error over 10 different training seeds for the RL and the CML methods. For the validation error, the shaded region shows the mean final position error over 100 trials for 10 different seeds.

To compare the computational complexity of the CML against that of the DQN (see Table 2), we first quantified the number of training steps each of them requires to solve the navigation problem. Employing a simplified reward (Eq. 6), the DQN converged to a maximum after approximately 60 K episode steps (Figure 4A, red), revealing the inherent complexity of the navigation task, i.e., the computational effort (number of steps) required to come up with a solution for the problem. Introducing a tailored reward (Eq. 7) increased this complexity, requiring about 130 K steps for convergence to account for the additional objective of the shortest path (Figure 4A, blue). In the case of the CML, the training error, measured as the difference between the internal estimate ${\hat{s}}_{t+1}$ and the actual value of the next state *s*_*t*+1_, kept decreasing even after 90 K steps (Figure 4B, dark green). However, the planning error, defined as the distance between the final position and the goal position during planning, converged after about 20 K steps (Figure 4B, light green), indicating that the CML can solve the problem at least as rapidly as the RL baseline.

**TABLE 2:** Comparison of the computational efficiency between the baseline DQN model and our CML.

Model	DQN	CML
Convergence (Number of steps)	∼60 K (Simplified reward)	∼90 K (Training error)
	∼130 K (Tailored reward)	∼20 K (Validation error)
**Parameters**	4874	4608
**Inference FLOPs**	9472	9464

As a secondary comparison metric, we quantified each architecture’s required parameters (see Section 2.4.3). Setting the size of the hidden layers to *N*_*h*1_ = *N*_*h*2_ = 64, the DQN network required 4874 parameters, while by setting the size of the embedding space *N*_*s*_ to 256 in the CML architecture, we constrained the required parameters to 4608. We chose the value of the hyperparameter *N*_*s*_ so that the total parameter count of our method matched that one of the RL baseline as closely as possible. Consequently, the CML demonstrated the ability to solve the navigation task without requiring more parameters than the DQN.

Finally, to compare the inference complexity of the two methods, we computed the number of floating point operations (FLOPs) per planning step. In our experiments, we set *N*_*i*_ to 2 and *N*_*α*_ to 8 for both networks. For the DQN, we used again *N*_*h*1_ = *N*_*h*2_ = 64 neurons per hidden layer, which resulted in requiring 9472 FLOPs per action selection. For the CML we set *N*_*s*_ to 256, resulting in a total number of 9464 FLOPs. This implies comparable efficiency between the two methods during planning. These comparisons suggest that the DQN and CML methods had similar computational complexity during training and planning for the navigation task.

To quantify the runtime requirements of our code (Table 3), we measured the average time required for the completion of one step during the exploration walks (training step) and one step during the goal-reaching task (inference step). We ran 100 exploration walks consisting of 900 steps each and averaged the step duration across them. We then ran 50 goal-reaching trials whose number of steps varied based on the distance between the randomly selected starting and goal positions and averaged the step duration across them too. We did this for both the random and the mixed exploration policy. All the experiments were performed on a 16-core AMD-Ryzen Threadripper PRO 3955WX CPU running at 2.2 GHz.

**TABLE 3:** Quantification of the computational complexity of the proposed method by evaluating its training and inference time.

Exploration strategy	Training step time (*μs*)	Inference step time (*μs*)
Random	99.776 ± 1.127	69.557 ± 2.773
Mixed	731.309 ± 336.876	71.835 ± 4.236

Notably, the local learning rule utilized by the CML model allows for in-place updates of the three parameter matrices during training. As a result, our method alleviates the need for the construction and storage of a computational graph for the model, decreasing the overall memory footprint.

### 3.2 Performance comparison against RL baseline

#### 3.2.1 Hyper-parameter evaluation

To assess the impact of different hyper-parameter choices on the performance of the CML in the navigation task, we conducted a systematic exploration by varying the number of exploration walks and the number of steps per walk.

**FIGURE 5:**
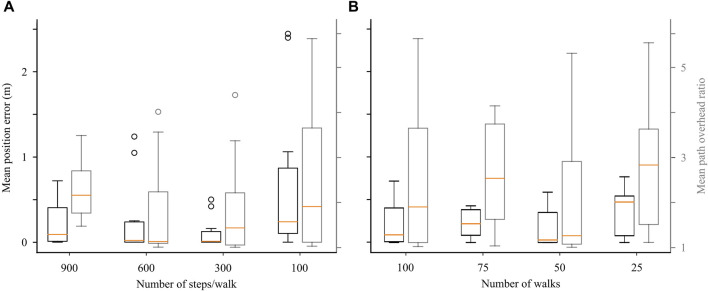
Exploring the effects of hyper-parameter choice. **(A)** Effect of decreasing the number of steps per random walk during training on final position error and path overhead ratio (Eq. 9) during planning. **(B)** Effect of decreasing the number of random walks during training on the planning position error and path overhead.

Initially, we trained the CML models with 100 random walks during exploration, with the number of steps per walk set to either 900 to 600, 300, or 100 (Figure 5A). We evaluated our models with respect to the final position error during planning. Intriguingly, an increase in the number of steps per walk did not consistently decrease the final position error, indicating a degree of overfitting. Notably, 300-step random walks demonstrated the best performance after 30 K training steps. Subsequently, we assessed the models with respect to the path overhead ratio, comparing the length of resulting navigation paths against optimal ones. Similar to the final position error, increasing the number of steps per walk did not consistently reduce the path length, with 600-step walks providing the best performance after 60 K total steps.

We then set the number of steps per walk to 900 and varied the total number of exploration walks, ranging from 100 to 75, 50, and 25 (Figure 5B). Evaluation based on the final position error during planning revealed that, again, a larger number of walks did not monotonically decrease the final position error, confirming the presence of possible overfitting. The models taking 50 random walks during exploration (45 K total training steps) achieved the best performance, a trend similarly observed in the path overhead ratio.

In conclusion, our results indicate the existence of a performance sweet spot, reached after approximately 45 K total training steps, suggesting that the hyper-parameter choice can affect the resulting performance.

#### 3.2.2 Mixed exploration strategy evaluation

To investigate the potential enhancement in navigation performance through a more sophisticated exploration, we extended the previous method with a mixed exploration strategy that combined novelty-based and random incentives. Specifically, we set the total number of walks to 100, with the last 70 being entirely random. In contrast, the first 30 walks were driven by a novelty-based incentive, encouraging the agent to explore unvisited locations within the environment. The impact of the novelty-based incentive gradually diminished through the 30 first walks (as per Eq. 5), incorporating random actions to revisit previously seen locations. After this novelty-driven exploration period, the strategy was entirely random.

**FIGURE 6:**
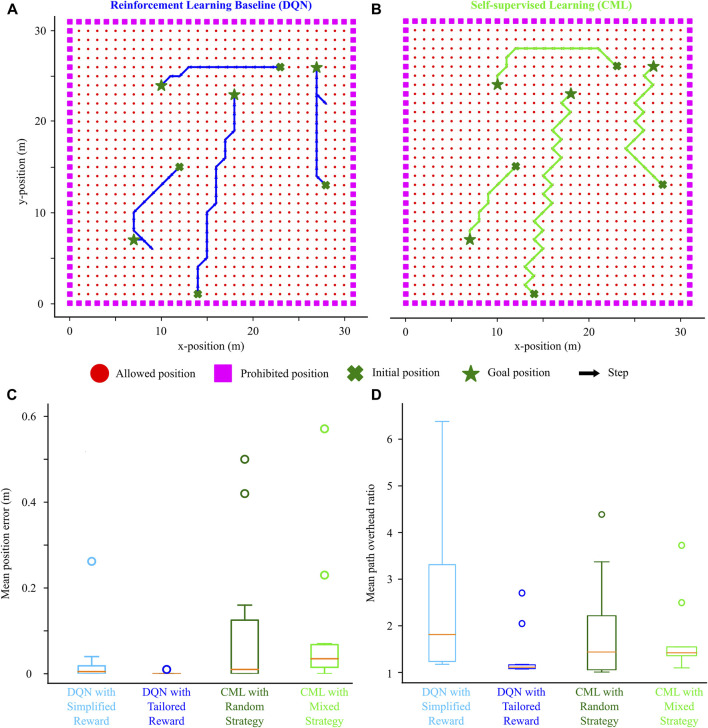
Effect of mixed exploration strategy (novelty-based and random) on the navigation performance. **(A)** Example trajectories from starting (cross) to initial (star) locations driven by the DQN. **(B)** Trajectories for the same starting-goal location pairs driven by the CML. **(C)** Comparison of the DQN (simplified and tailored reward) against the CML (random and mixed exploration strategy) based on final position error. **(D)** Similar comparison based on the path overhead ratio.

The mixed and random exploration strategies provided similar navigation performance, comparable with the DQN baseline (Figure 6). Trajectories generated by both methods exhibited some variability but effectively guided the agent from the initial (cross) to the goal (star) locations. Although the mixed exploration strategy yielded a slightly better final position error than the random strategy, both were comparable to that of the DQN with a simplified reward. As expected, the DQN with the tailored reward outperformed other methods, partly attributed to its extended training session. The benefit of the mixed exploration strategy emerged when evaluating the path overhead ratio. Following training with the mixed exploration strategy, CML models not only outperformed the DQN using the simplified reward but also performed comparably to the DQN with the tailored reward without requiring prolonged training sessions. Consequently, our results suggest that the novelty-based exploration factor, fostering the exploration of new locations during training, enables the method to identify shorter paths between initial and goal locations, providing some performance improvement without additional training.

### 3.3 Generalization to larger environments

To investigate the potential benefits beyond path length improvement, we hypothesized that the more thorough coverage of the environment promoted by novelty-based exploration could enhance the generalization of the method, especially when dealing with larger environments where previously unvisited locations arise often. To test this hypothesis, we trained CML models utilizing both random and mixed exploration strategies within a 30 × 30 m arena. Subsequently, we evaluated the trained models not only in the training arena but also in larger environments of 60 × 60 m and 120 × 120 m.

**FIGURE 7:**
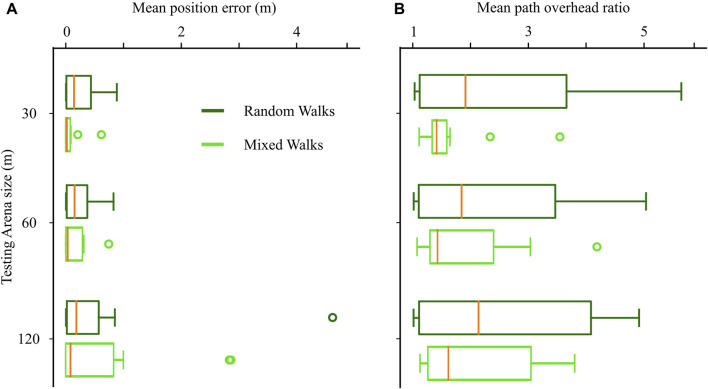
Effect of mixed exploration strategy (novelty-based and random walks) on the navigation in unknown environments larger than the training arena. **(A)** Final position error in arenas of increasing size. **(B)** Path overhead ratio in arenas of increasing size.

The models trained with the mixed exploration strategy exhibited superior navigation performance compared to those trained with the random strategy (Figure 7). This improvement was reflected in the lower median values for both the final position error and the path overhead ratio when employing the mixed navigation strategy. These results support our hypothesis that the more extensive exploration facilitated by the mixed strategy contributes to enhanced generalization, particularly in larger environments where novel locations are more frequent.

### 3.4 Generalization to unknown cluttered environments

**FIGURE 8:**
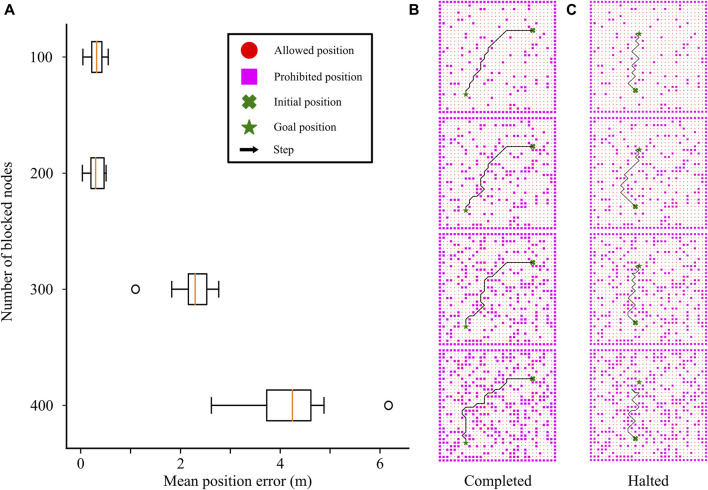
Effect of uncharted obstacles on the navigation performance. **(A)** Final position error as a function of the number of obstacles (blocked nodes) in the action space. **(B)** Successful case: CML leading from starting (cross) to final (star) positions as the number of obstacles increases from 100 (top panel) to 400 (bottom panel). **(C)** Failure case: CML successfully leading from initial (cross) to goal (star) positions with 100, 200, and 300 obstacles, but halting in the presence of 400 (bottom panel).

The ability of the CML models to navigate larger, unseen environments made us wonder whether they could handle other types of variability in the environment during planning. For this, we examined whether our CML models could navigate through environments cluttered with obstacles that had not been experienced during training (Figure 8). We first trained CML models in a 30 × 30 m arena using a random exploration strategy. Subsequently, we assessed the models’ performance in navigating from an initial (cross) to a goal (star) location in an environment of the same dimensions but with an increasing number of obstacles (Figures 8B, C). Specifically, we introduced 100, 200, 300, and 400 point-obstacles randomly placed in the environment, rendering specific locations impassable for the robot. Since the task involved finding any path between the initial and goal locations, optimizing path length by the mixed exploration strategy was not deemed crucial. Considering the additional computational complexity the mixed exploration policy introduces, we opted to train our CML models using only the random exploration strategy.

The trained CML models successfully navigated environments with 100 and 200 obstacles, as evidenced by the small final position errors (Figure 8A). However, challenges arose as the complexity of the environment increased with 300 or 400 obstacles. The final position error increased with the number of obstacles, attributed to failure cases where the agent approached the goal positions but halted without reaching them (Figure 8C, bottom). This behavior demonstrated the model’s limitations in handling complex scenarios with increased obstacle density, which is characteristic of local planners lacking global knowledge of the environment.

### 3.5 Application to continuous spaces

Lastly, we examined the applicability of our method to real-world navigation tasks within continuous 2D spaces. For this, we simulated a wheeled robot within a square 30 × 30 m arena and trained a CML model to govern its navigation. Throughout the training, the robot employed the random exploration strategy, engaging in exploratory walks throughout the arena. Then, the trained model planned the robot’s navigation.

**FIGURE 9:**
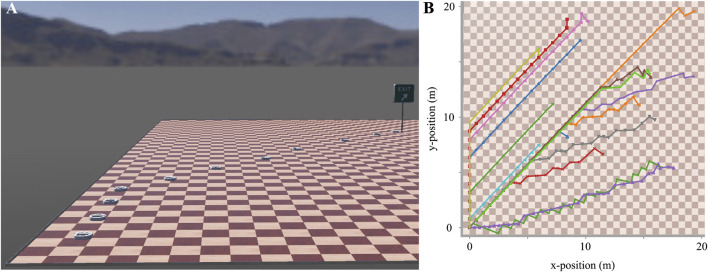
Example trajectories of the wheeled robot when navigating in the continuous 2D arena from an initial base position (bottom left) to random goal locations. **(A)** Intermediate positions of the robot while navigating from the initial position (bottom left) to the goal location (exit sign). **(B)** Multiple trajectories like the one shown in A demonstrate the paths chosen by the CML.

During planning, we set the initial position of the robot without loss of generality to an arbitrary base location (Figure 9A, bottom left) and generated random goal locations 9, A, exit sign) on the 2D plane of the arena. Considering the continuous nature of the 2D locations, the simulation halted either when the robot’s position was within a circle with a radius of 0.25m around the goal location or after a maximum of 100 actions had been taken. An illustrative trajectory of the robot navigating the arena is presented in Figure 9. As it becomes evident from the trajectories shown in Figure 9B, the paths taken during different iterations did not precisely follow straight lines from the initial to the goal locations.

Consequently, while the CML introduced some sub-optimality in the path planning, it consistently demonstrated successful navigation in the 2D space. Despite the deviations from optimal paths, the CML showcased its adaptability to real-world scenarios, emphasizing its robustness in handling continuous and dynamic 2D navigation tasks.

## 4 Discussion

In this work, we focused on expanding the brain-inspired planning method of Cognitive Map Learners (CML) (Stöckl et al., 2022) to address the 2D navigation of mobile agents at the edge. Overall, our findings underscore the potential of embodied neuromorphic agents, showcasing robust performance with minimal resource utilization at the edge.

In terms of computational complexity for both training and inference, our proposed CML was directly comparable to the DQN baseline. The CML exhibited convergence to a network architecture capable of guiding the robot from any initial to a goal location at least as fast as the DQN baseline. Interestingly, a DQN with a reward tailored to the optimal solution of the task demanded additional training episodes, while its performance was challenged by the same CML architecture extended with a more elegant, novelty-driven exploration strategy. Both models had similar numbers of variable parameters and required comparable numbers of FLOPs per inference step. However, the CML model allowed for in-place weight updates and did not necessitate additional computational graphs during training (for error backpropagation), resulting in a reduced memory footprint.

The self-supervised nature of the method is a critical feature that simplifies the training of agents at the edge, in contrast to the well-established RL methods that require intricate rewards that are well-tuned and carefully tailored to specific tasks. Using the deviation between the estimated and the actual next state, the CML uses only an internal state to improve its performance without focusing on externally provided rewards. While this simplifies the training, it requires a clear definition of the task objective in the agent’s state; otherwise, it may lead to suboptimal solutions. For example, in our case, the lack of encoding of the path length in the agent’s state resulted in deviation from the shortest ones. However, the simplicity of the method that allows for its seamless application to any state-defined agent operating in discrete action spaces can be preserved, while improving the optimality of the planning with more elegant exploration strategies.

More specifically, the exploration strategies employed in our method that expanded the simplistic random walks of the original approach with a novelty-driven factor contribute to a closer-to-optimal planning performance. These strategies introduce inevitable computational requirements, such as the nearest-neighbor storage and selection during novelty-based exploration, without affecting the baseline performance of the method. As a result, they introduce a balance between performance optimality and resource efficiency that can be adapted based on the task and the available resources.

With this primary objective of resource efficiency, we also extended the method and validated it in dynamic environments featuring unmapped obstacles. To do so, we deliberately did not integrate distance sensor readouts in the embedding space. Instead, when the agent planned a movement into a prohibited location corresponding to an obstacle, we marked this action as not affordable and modified the decision of the model. The CML did not aim to encode the obstacle locations and memorize how to avoid them but encountered them during planning and tried to find alternative paths reactively. This design choice reduced the dimensionality of our model and allowed our algorithm to readily adapt to different obstacle placements during planning. The performance was competitive while utilizing minimal resource requirements, a crucial aspect for edge applications. However, being only a local planner, our method did give rise to halting scenarios, where the agent gets trapped in between obstacles and cannot find a path to the goal location although such may exist if additional steps are taken to bypass the prohibited locations.

## 5 Limitations and future work

While the performance of our method is competitive, it still demonstrates some crucial limitations that leave room for future work.

First, our adaptation of the CML architecture to the 2D planning task leads naturally to the possibility of expanding to three dimensions. Practically, the proposed method can directly scale up to three dimensions to address the planning of actions for agents with such capabilities. The resource requirements of such an upscaling would increase proportionally to with the number of dimensions to encode the new dimension into the state embedding. However, the efficiency of the method would be even more crucial for agents planning in three dimensions, such as drones with minimal onboard resources.

A second limitation emanating from the size of the embedding space affects planning in cluttered environments. In our model, we deliberately did not integrate distance sensor readouts in the embedding space. Despite the competitive performance of the presented method, we would like to explore in future work whether this extension could address the halting cases when the number of environmental obstacles increases.

A third limitation emerges due to the nature of the action space. Similarly to the original CML model, we focused only on discrete sets of possible actions. In future work, we aim to address this limitation in two ways. First, we want to combine a CML acting as a high-level planner with one or more low-level planners. Such could be bioinspired controllers that require no or minimal training to drive the low-level behaviors through multiple degrees of freedom (Ijspeert et al., 2007; Polykretis et al., 2020; Polykretis et al., 2023). In the long term, we would also like to explore the extension of the CML to continuous action spaces to address different sets of tasks.

Lastly, while we designed our method with the requirements of edge hardware in mind, an implementation on such a device is missing. Unlike many existing methods that are trained offline and allow for inference on neuromorphic hardware (Tang et al., 2020; Taunyazov et al., 2020; Stewart et al., 2023), local learning rules theoretically extend their applicability to neuromorphic processors during the training phase. The local learning rule that alleviates the need for backpropagation renders CML compatible with on-chip training while introducing a minimal memory footprint since weight updates can be done in-place. Promising intermediate results have already been presented for Intel’s Loihi2, but memristive crossbar arrays (Strukov et al., 2008), capable of performing matrix-vector multiplications at constant complexity (Truong and Min, 2014; Assaf et al., 2018) are an even more promising fit for such an implementation, guiding us to our next steps.

## Data Availability

The raw data supporting the conclusion of this article will be made available by the authors, without undue reservation.
